# Illumination‐Dependent Interfacial Recombination in Perovskite Photovoltaics Driven by Trap States in Metal Oxide Electron Transport Layers

**DOI:** 10.1002/advs.78501

**Published:** 2026-10-30

**Authors:** Tianhao Lan, Joel Luke, Oskar J. Sandberg, Charlie Henderson, Ying Sun, Katherine Stewart, Ji‐Seon Kim

**Affiliations:** ^1^ Department of Physics and Centre for Processable Electronics Imperial College London London UK; ^2^ Physical Sciences and Engineering Division (PSE) King Abdullah University of Science and Technology (KAUST) Thuwal Saudi Arabia; ^3^ Department of Physics Faculty of Science and Engineering Åbo Akademi University Turku Finland; ^4^ Department of Chemistry University of Oxford Oxford UK; ^5^ Department of Physics Ewha Womans University Seoul Republic of Korea

**Keywords:** charge carrier, electron transport chain, energy conversion efficiency, materials science, metal, optoelectronics, oxide, perovskite, photovoltaic system, photovoltaics

## Abstract

Perovskite solar cells (PSCs) now exceed 27% power conversion efficiency (PCE); however their performance under varying illumination conditions remains strongly influenced by interfacial charge carrier dynamics. Although bulk defects have been substantially reduced through perovskite compositional engineering, trap states in metal oxide electron transport layers (ETLs) can still capture carriers and promote non‐radiative recombination, with effects that depend strongly on photon flux. Here, we investigate trap‐assisted interfacial recombination in metal oxide ETLs using a Low‐High‐Low (LHL) illumination protocol, which probes device behavior from low‐light (1000 lux) to 1 Sun and back. We find that interfacial trap states have only a minor effect under low‐light but become highly detrimental under 1 Sun illumination, where they dominate interfacial recombination and reduce the open‐circuit voltage (V_OC_). This effect is particularly pronounced for metal oxides with higher densities of surface trap states, such as TiO_2_. Upon returning to low‐light after 1 Sun exposure, devices show a marked V_OC_ decrease followed by slow recovery, consistent with gradual charge release from interfacial traps and the subsequent suppression of recombination. These findings reveal the strongly illumination‐dependent role of metal oxide trap states and provide design guidance for optimizing PSCs for both outdoor and indoor photovoltaic applications.

## Graphical abstract

Changes in light intensity impact trap states introduced at the metal oxide/perovskite interface. Comparing SnO_2_, TiO_2_ and ZnO electron transport layers reveals high interfacial trapping and recombination under high‐light, while bulk defects become more dominant under low‐light. These findings provide important device design rules required for different illumination conditions.

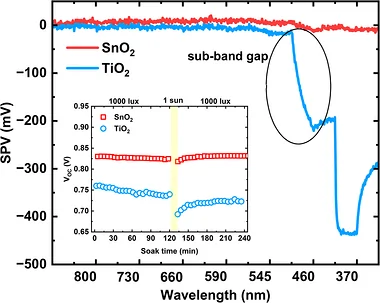

## 1 Introduction

Perovskite solar cells (PSCs) have emerged as leading candidates in photovoltaic technology, achieving certified power conversion efficiencies exceeding 27% and showing strong potential for outdoor and indoor photovoltaics [1, 2]. In such applications under varying light intensities, charge dynamics play a key role in determining device performance and stability. Although compositional engineering of the perovskite photoactive layer has produced effectively better structural stability and reduced defect densities [1, 3], controlling charge dynamics across different light intensities remains a challenge, particularly at interfaces between the perovskite layer and the charge transport layers [4].

For the conventional n‐i‐p structure, many inorganic materials with good crystallinity and morphology, such as metal oxides, have been developed as n‐type interlayers to meet the requirements for perovskite deposition [5, 6]. Despite compatible energy level alignment between commonly used ETLs and the perovskite layer, interfacial trap states can capture photogenerated carriers, thereby facilitating non‐radiative recombination and reducing V_OC_ and fill factor (FF) [7, 8, 9]. These losses predominantly manifest at interfacial regions between the perovskite layer and ETL in the n‐i‐p structure [10]. To mitigate this energy loss, passivation layers have been introduced to reduce interfacial defects and provide a better surface for perovskite deposition [4, 8]. However, such approaches often add processing complexity, increase series resistance, or inhibit thermal and environmental durability [11, 12, 13].

Metal oxide layers have been widely employed as electron transport materials in PSCs. Their smooth surface and high carrier mobility have enabled significant performance improvements. Despite this, metal oxides possess defects, which can impact electron transport and extraction. Previous studies have reported that the donor‐like behavior of metal oxide defects such as oxygen vacancies contributes to the n‐type conductivity by releasing free electrons into the conduction band. However, while oxygen vacancies in the bulk can be beneficial for conductivity, trap states near the surface can drive trap‐assisted recombination when contact with other layers. A previous report also revealed a correlation between metal oxide trap state‐induced interfacial band bending and the light‐soaking effect of photovoltaic devices [14]. These observations imply a fundamental trade‐off between improved conductivity by metal oxide defects and charge extraction [14]. Therefore, it is crucial to find an effective method for the trap states/defects modification on the metal oxide surface and understand the charge dynamics related to trap states at the interface. So far, the majority of studies are performed under standard test conditions (1 Sun, AM1.5G, 100 mW cm^−2^) [15] and are not sufficient to understand the performance of devices for low‐intensity indoor applications (100–1500 lux) [16]. A more systematic, illumination‐dependent investigation is therefore required to clarify the relationship between metal oxide ETL defects and device performance under varying light intensity conditions.

In this study, commonly used metal oxides with different trap state densities are used to investigate how illumination‐dependent charge dynamics induced by ETL trap states affect the performance of perovskite solar cells. To simplify the interpretation, we use high‐light and low‐light to refer to outdoor light conditions (3–100 mW/cm^2^, 0.03‐ 1 Sun equivalent) and indoor light conditions (500‐ 2500 lux). We demonstrate that the negative effect of trap states on the device performance becomes more detrimental under high‐light. Under low‐light, the interfacial recombination loss induced by metal oxides is largely suppressed due to lower charge carrier density. Surface photovoltage (SPV) measurements on metal oxide ETLs illustrate distinct difference in photo‐response induced by surface trap states, and this is correlated with the difference in non‐radiative recombination observed in light intensity dependent variation of devices. To probe charge behavior related to ETL trap states, we conduct a Low‐High‐Low (LHL) light intensity exposure experiment: beginning at low‐light (1000 lux), ramping to high‐light (100 mW cm^−2^, 1 Sun equivalent), and returning to low‐light (1000 lux). This protocol reveals how charge trapping and recombination are affected by metal oxide trap states and influence device parameters. Specifically, the observed drop in V_OC_ upon 1 Sun illumination indicates that trap states in metal oxide ETLs increase interfacial recombination when they are activated by the high‐light, while under low‐light this interfacial recombination is suppressed, highlighting the different optimization considerations required for different light intensities.

## 2 Results and Discussion

### 2.1 Characterization of ETL Trap States

The surface electronic response of the metal oxide ETLs is first characterized by the surface photovoltage (SPV) technique. In the SPV measurements, a change in contact potential difference (CPD) upon illumination is measured, which reflects a change in the surface potential or work function. This change is caused by the redistribution of photogenerated charges near the surface, where the electron and hole move toward different directions under the influence of band bending, surface charges, and trap states [17]. For typical n‐type metal oxides, intrinsic defects such as atomic vacancies, interstitials and adsorbate‐related states can trap charge under illumination, acting as surface trap states and producing a photo‐induced surface potential change, which corresponds to the measured SPV. As such, the SPV response under sub‐bandgap excitation indicates the presence of trap states within the bandgap. In this work, this SPV magnitude is interpreted as a measure of photo‐response associated with surface trap states, and the charge dynamics are indicated by SPV transient. For instance, a larger SPV turn‐on magnitude is indicative of a relatively larger amount of surface trap states that induce a significant change in surface potential upon illumination, while the slow changing rate of SPV suggests gradual trap filling or a broad trap energy distribution. After the light is switched off, fast recovery indicates reversible charge redistribution, whereas slow or incomplete recovery points to long‐lived trapped charges or irreversible chemical changes [18].

We monitored the SPV response over three cycles of alternating light‐dark conditions (100s light/150s dark) under a white light (Figure 1a, Figure S1a). SnO_2_ exhibits minimal SPV response, whereas TiO_2_ exhibits a much larger SPV response, reaching more than −200 mV at the end of the first illumination period, followed by only partial recovery in the dark. This slow SPV response under alternating light‐dark cycles illustrates that charge relaxation is slow in TiO_2_ [19, 20]. As a further comparison, ZnO, another n‐type metal oxide commonly employed as an ETL in optoelectronic devices, was measured under the same conditions. ZnO displays a large SPV up to −300 mV under white light and also limited recovery in the dark, which was similar with the previous KPFM‐SPV measurements, illustrating that both large SPV magnitude and slow decay are induced by surface trap states and oxygen desorption/relaxation processes [21, 22]. SPV results are consistent with long‐lived trapped charge and slowly reversible adsorbate‐related surface charging found in ZnO surfaces [23]. The smallest magnitude of SnO_2_ suggests the fewest such long‐lived photo‐charged states under the same measurement conditions compared to TiO_2_ and ZnO [19, 20].

**FIGURE 1:**
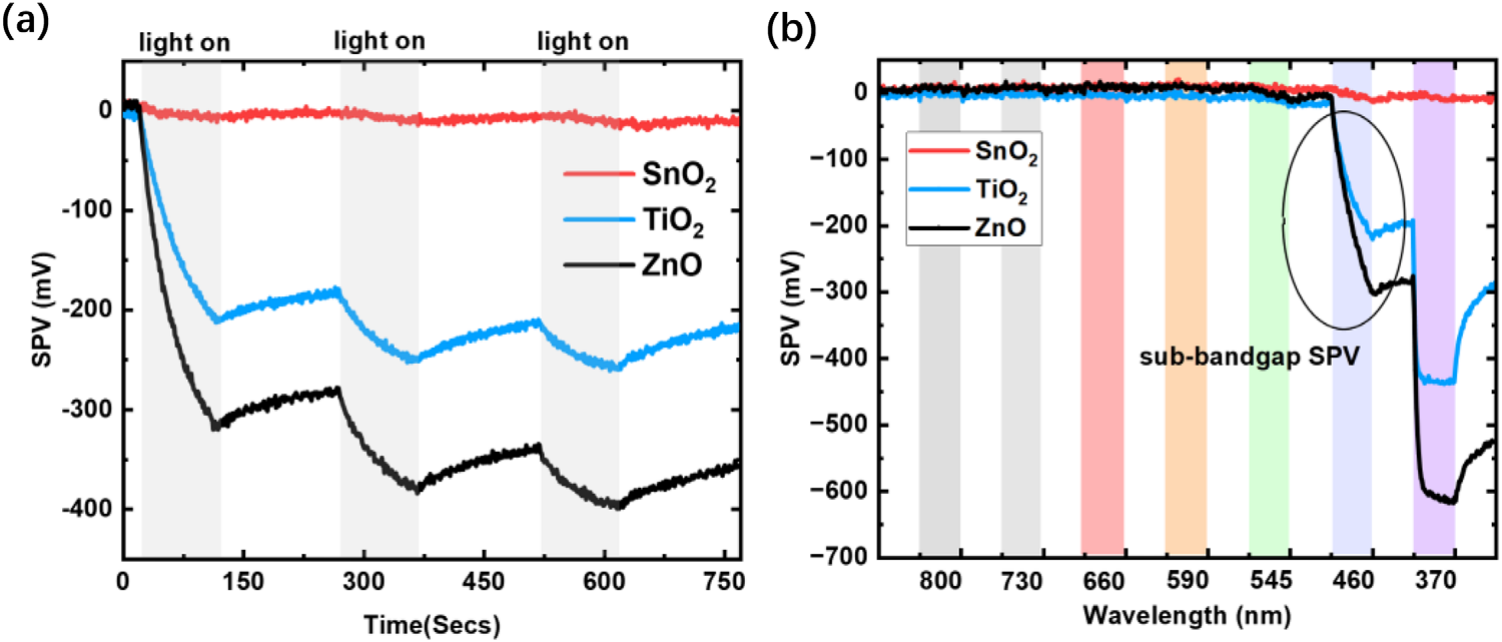
(a) SPV measurements (initial value set to 0) of TiO_2_ and SnO_2_ thin films under white light. (b) Wavelength‐dependent SPV measurements of different metal oxides under monochromatic LED illumination. Highlighted regions indicate light illumination. All samples are the ETL only without the perovskite layer. Bandgaps for SnO_2_, TiO_2_ and ZnO are 3.6, 3.1 and 3.3 eV, respectively [15, 28].

To further examine how trap‐band absorption contributes to the SPV response, wavelength‐dependent SPV measurements were conducted using monochromatic LEDs (Figure S1b). As shown in Figure 2b, TiO_2_ shows clear SPV responses at both 460 and 370 nm. Since 460 nm is below the bandgap of TiO_2_ [15, 24], this response indicates that trap‐induced surface potential change makes a significant contribution to the SPV. In contrast, SnO_2_ shows only a weak response at both 460 and 370 nm. This indicates that trap state‐induced surface potential is minimal within the photon‐energy range probed here. Together with the weak response under the white light, the SPV results illustrate that SnO_2_ shows a stable surface potential across the whole illumination spectrum, which is consistent with previous study of UV stability for SnO_2_ due to its wide bandgap [25, 26]. To investigate the impact of surface adsorbates, we measured the SPV response of the oxides under study in high vacuum (∼10^−5^ mbar) conditions (Figure S2). The magnitude of SPV of both SnO_2_ and TiO_2_ become larger under high vacuum, and the relative relationship between their SPV remain the same trend except ZnO. Our results are consistent with previous study that illustrated a lower density of traps in SnO_2_‐based devices than TiO_2_‐based devices [25, 26]. As for ZnO, an even stronger sub‐bandgap photo‐response was observed at 460 nm, suggesting a relatively larger amount trap states at the ZnO surface compared to both TiO_2_ and SnO_2_. It is noted that devices based on ZnO ETL showed much poorer performance and most pixels were dead after the first scanning, suggesting such surface trap states significantly worsen the interface quality. This is consistent with widely reported strong photocatalytic performance and poor thermal stability [27]. In summary, these findings show that SnO_2_ has fewer trap states than TiO_2_ and ZnO, indicating a more stable interface between the SnO_2_ ETL and the perovskite layer in the photovoltaics. An energy band schematic of the metal oxide measured under sub‐bandgap excitation is shown in Figure S3.

**FIGURE 2:**
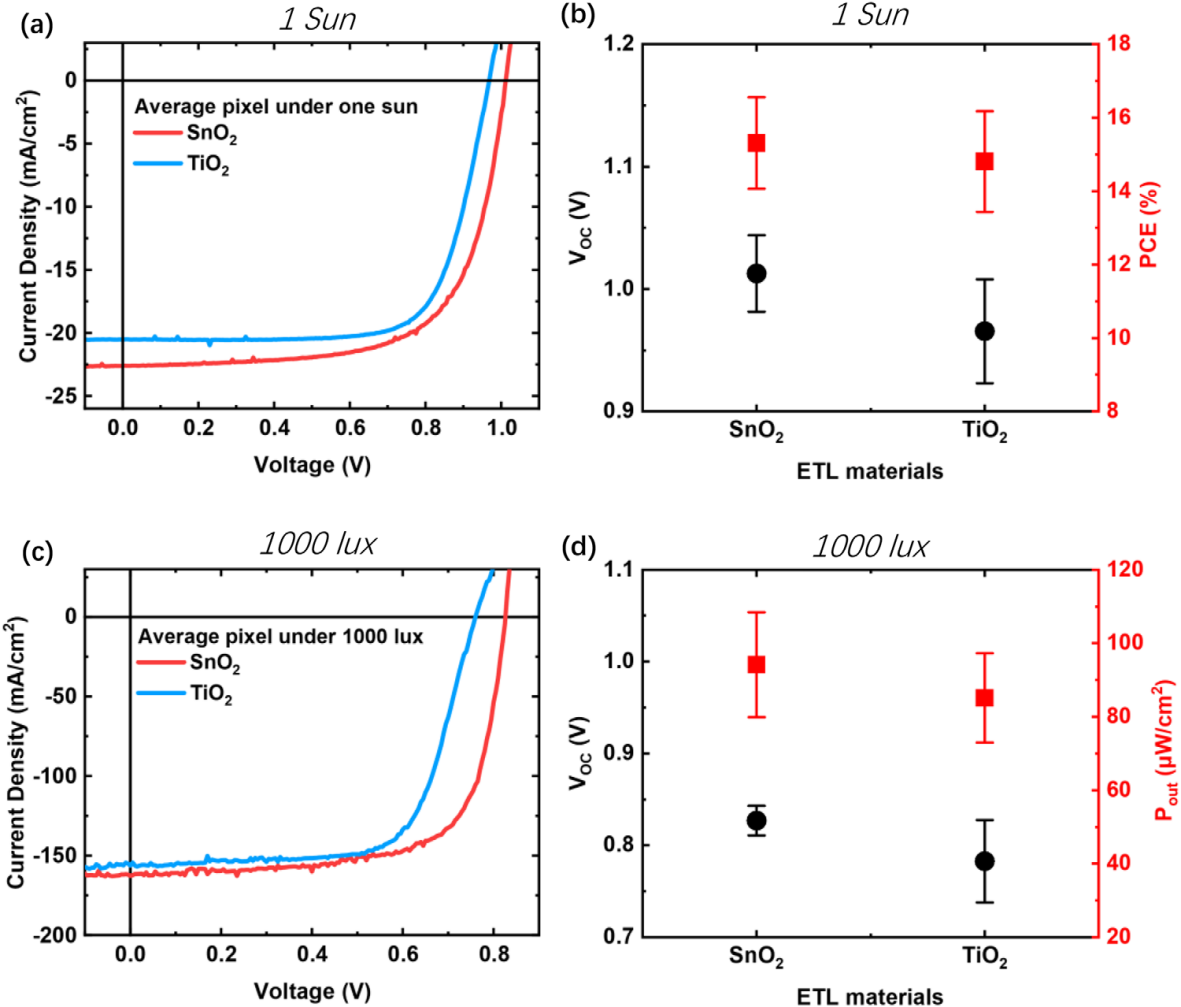
(a) J‐V characteristics, (b) V_OC_ and PCE average values of perovskite solar cells using different metal oxides as the ETL under 1 Sun. (Device number > 27) (c) J‐V characteristics, (d) V_OC_ and peak power output average values measured under 1000 lux. (Device number > 8).

### 2.2 Impact of ETL Trap States on Device Performance

To investigate how ETL trap states influence PSC performance, n‐i‐p devices based on different metal oxide ETLs were fabricated to compare changes in optoelectronic properties. A mixed cation/anion perovskite with a stoichiometry of Cs_0.05_Ma_0.14_FA_0.81_Pb(I_0.86_Br_0.14_)_3_ and 2,20,7,70‐tetrakis[N, N‐di(4‐methoxyphenyl)amino]‐9,90‐spirobifluorene (Spiro‐OMeTAD) are employed as the active layer and the hole transport layer (HTL). By using bare metal oxides without treatments or passivation layers, the difference of device performance directly illustrates the influence of metal oxide traps. SnO_2_, TiO_2_ and ZnO are used as the ETLs in this work. SPV measurements of perovskite layers on top of the metal oxides confirmed their electron selectivity (Figure S4) [29].

Figure 2a presents the J‐V characteristics of the average device with different metal oxide ETLs. Under high‐light (1 Sun), SnO_2_‐based devices show a higher open‐circuit voltage (V_OC_) and current density (J_SC_) than TiO_2_‐based devices. The higher V_OC_ and J_SC_ in the SnO_2_‐based devices indicate SnO_2_’s suppressed recombination loss. Although a higher fill factor (FF) can be achieved by TiO_2_, the power conversion efficiency (PCE) is still lower than SnO_2_‐based devices due to lower V_OC_ (Figure 1b). Given that all layers except the ETL are the same in the devices, this performance difference is only due to the electron transport ability of metal oxides and the interface between the ETL and the perovskite layer, which is crucial for comparing the impact of metal oxide traps in the following experiments. From the J‐V characteristics, it is found that V_OC_ follows the same trend as the amount of surface trap states we confirmed in the above SPV measurements. To extend this comparison to more metal oxide ETLs devices are also fabricated based on ZnO, a commonly used ETL in organic photovoltaics [30]. Despite the same device structure, the performance of ZnO‐based devices is poorer compared to that of TiO_2_ and SnO_2_ devices, mainly due to lower V_OC_ (Figure S5). This significant V_OC_ loss is attributed to more trap states associated with by‐products during ZnO formation and its chemical instability with the perovskite [18, 27, 31].

To investigate whether the metal oxide ETL‐induced trap states influence device performance, further J‐V characteristics are measured under low‐light. Under low‐light (1000 lux), all devices show poorer V_OC_ and J_SC_ than those at 1 Sun due to the reduced photon flux, but the relative relationship of V_OC_ between ETLs remains the same (Figure 2c), indicating that V_OC_ loss induced by metal oxide ETLs exists under varying light conditions. Like the case under high‐light, the peak power output (P_out_) measured under 1000 lux of TiO_2_‐based devices is lower than SnO_2_‐based devices due to lower V_OC_ despite higher FF, suggesting that device performance is significantly dependent on the V_OC_ under varying light conditions (Figure 2d). The statistical distributions of V_OC_, J_SC_, FF and PCE/P_out_ are displayed in Figure S5, while Table S1 provides a summary of the key device parameters.

To investigate further the impact of metal oxide trap states on the recombination mechanism of perovskite photovoltaics, the light intensity dependent variation of V_OC_ and J_SC_ is analyzed. Previous research has attributed the slope in a semi‐log plot of V_OC_ as a function of light intensity (*I*) as indicative of a particular mechanism dominating recombination loss with the formula *V_OC_ = nkTln(I)/q* [32]. More specifically, the Ideality Factor, *n*, extracted from the slope of semi‐log plots should be 1 in the case of an ideal diode where only second‐order radiative (bimolecular) recombination is considered, whereas if *n* equals 2 it suggests first‐order (monomolecular) non‐radiative recombination is dominant [32]. More specifically, the Ideality Factor, *n*, extracted from the slope of semi‐log plots should be 1 in the case of an ideal diode where only second‐order radiative (bimolecular) recombination is considered, whereas if *n* equals 2 it suggests first‐order (monomolecular) non‐radiative recombination is dominant. The *n* between 1 and 2 suggests a mixture of radiative and non‐radiative recombination [33]. Figure 3a shows that under high‐light (3–100 mW/cm^2^), the V_OC_ of SnO_2_ linearly increases with a slope of 1.045 kT/q, close to the ideal radiative recombination limit (1 kT/q), indicating dominant radiative recombination and minimal trap‐assisted non‐radiative recombination. Previous work has suggested that a first‐order non‐radiative trap‐assisted recombination is dominated by processes occurring at the interfaces, while second‐order radiative recombination is believed to originate from the bulk of the perovskite absorber, indicating that first‐ and second‐order recombination processes are controlled by different carrier reservoirs [33]. In this case, the Ideality Factor is possible to be smaller than unity in the presence of strong interfacial recombination [34]. Similar non‐ideal behavior has been reported in some real devices [35]. Also, these interfacial trap states can capture photogenerated carriers and form local space charge. Previous study has shown that interfacial charge accumulation can redistribute or screen the internal electric field in perovskite solar cells [36]. Charge accumulation at buried interfaces has also been shown to modify the effective carrier extraction barrier, and it was associated with perovskite ions and vacancies [37]. Given that the same perovskite are used across our study, the ion migration is affected only by the internal electric field. Therefore, the sub‐unity Ideality Factor observed in the TiO_2_‐based device (0.760 kT/q) is not assigned to non‐radiative recombination in the bulk of the perovskite. Instead, it suggests a significant interfacial trap‐assisted recombination loss and non‐ideal electrostatic conditions induced by TiO_2_ compared to SnO_2_, which is consistent with our SPV and simulation results.

**FIGURE 3:**
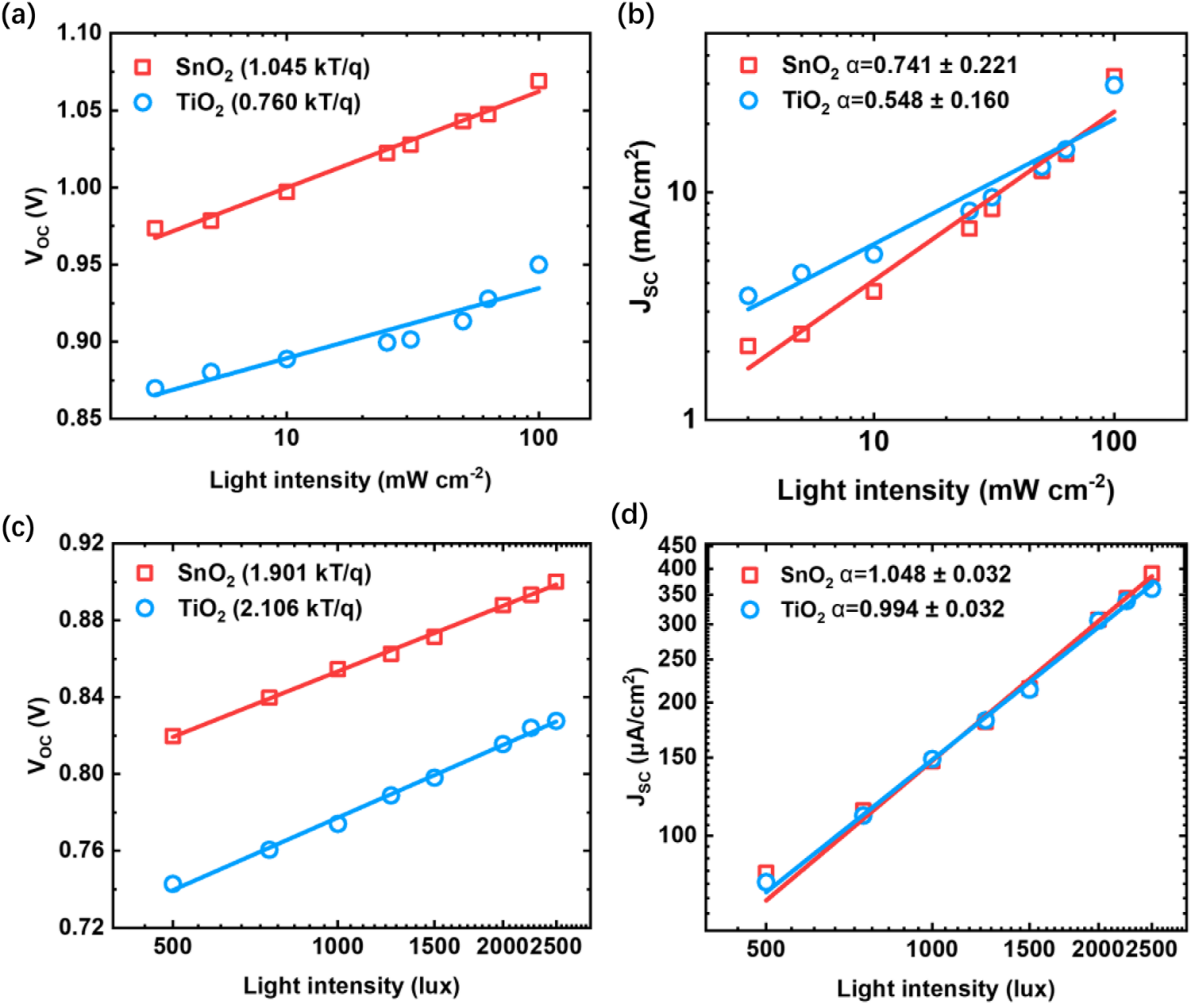
Light intensity‐dependent V_OC_ and J_SC_ under (a, b) high‐light and under (c, d) low‐light.

The J_SC_ as a function of light intensity analysis was also performed to examine the degree of bimolecular recombination under high‐light in SnO_2_ and TiO_2_‐based devices (Figure 3b). The slopes were fitted based on the power law relationship between J_SC_ and light intensity *I* (J_SC_ ∝ *I^α^*), where *α* = 1 in the case of negligible bimolecular recombination [38]. The slope of the linear J_SC_ variation with respect to light intensity in a log‐log scale was 0.741 and 0.548 in SnO_2_ and TiO_2_‐based devices, respectively. The slope *α* lower than 1 suggests significant bimolecular recombination with both ETLs, with TiO_2_ devices having the shallower gradient indicating stronger bimolecular recombination than SnO_2_‐based devices.

Under low‐light (500–2500 lux), the V_OC_ of both devices increases linearly with light intensity, with similar slopes of 1.901 and 2.106 kT/q for SnO_2_ and TiO_2_ devices, respectively, indicating dominant non‐radiative recombination, in which electrons and holes recombine via defects or traps. The slopes of the light intensity‐dependent J_SC_ for SnO_2_‐ and TiO_2_‐based devices are both close to 1, indicating that bimolecular recombination is not a problem under low‐light. The similar slope of light intensity‐dependent J_SC_ and V_OC_ in the SnO_2_ and TiO_2_ devices under low‐light indicates that non‐radiative recombination loss is mainly induced by trap states in the bulk of the perovskite.

These findings demonstrate that SnO_2_’s superior performance arises from better electron transport and suppressed non‐radiative recombination, as evidenced by light intensity dependence analysis of photovoltaic parameters, while TiO_2_’s limitations arise from non‐radiative recombination, which is particularly pronounced under high‐light. However, non‐radiative recombination loss is dominated by bulk processes in the perovskite under low‐light, rather than interfacial recombination. Light intensity‐dependent variation shows that using metal oxide ETLs with fewer trap states is crucial for suppressing interfacial recombination and thereby improving device V_OC_ under high‐light, without sacrificing perovskite film quality on substrates (Figures S6 and S7), which might be the main concern in indoor photovoltaics.

For further analysis, we set up a Low‐High‐Low (LHL) light protocol to measure J‐V characteristics at varying light intensities and evaluate the light‐intensity dependence of ETL‐induced interfacial recombination. In this study, we conducted stability tests under low‐light with an exposure under high‐light for a short time (2 h at 1000 lux – 10 min at 1 Sun – 2 h at 1000 lux) to follow how V_OC_, J_SC_, FF, and PCE evolve when carrier density is changed (Figure 4). Devices with SnO_2_ exhibited stable J‐V characteristics during the full cycle. During the first 2 h at 1000 lux, the V_OC_ decreased very slightly from 0.830 to 0.824 V (99.3% retention). After being illuminated under 1 Sun for 10 min, the V_OC_ at 1000 lux dropped slightly to 0.818 V before recovering to 0.831 V during the second low‐light step. In contrast, TiO_2_‐based devices showed more initial V_OC_ loss under low‐light, falling from 0.760 to 0.740 V (97.4% retention) after 2 h followed by a sharp drop to 0.692 V after the 1 Sun step. During the second 2 h at 1000 lux, V_OC_ recovers only partially to 0.722 V, indicating a partial, permanent loss caused by the high‐light step. Across the cycle, J_SC_ remained relatively stable for both ETLs, while FF and PCE largely tracked V_OC_ changes.

**FIGURE 4:**
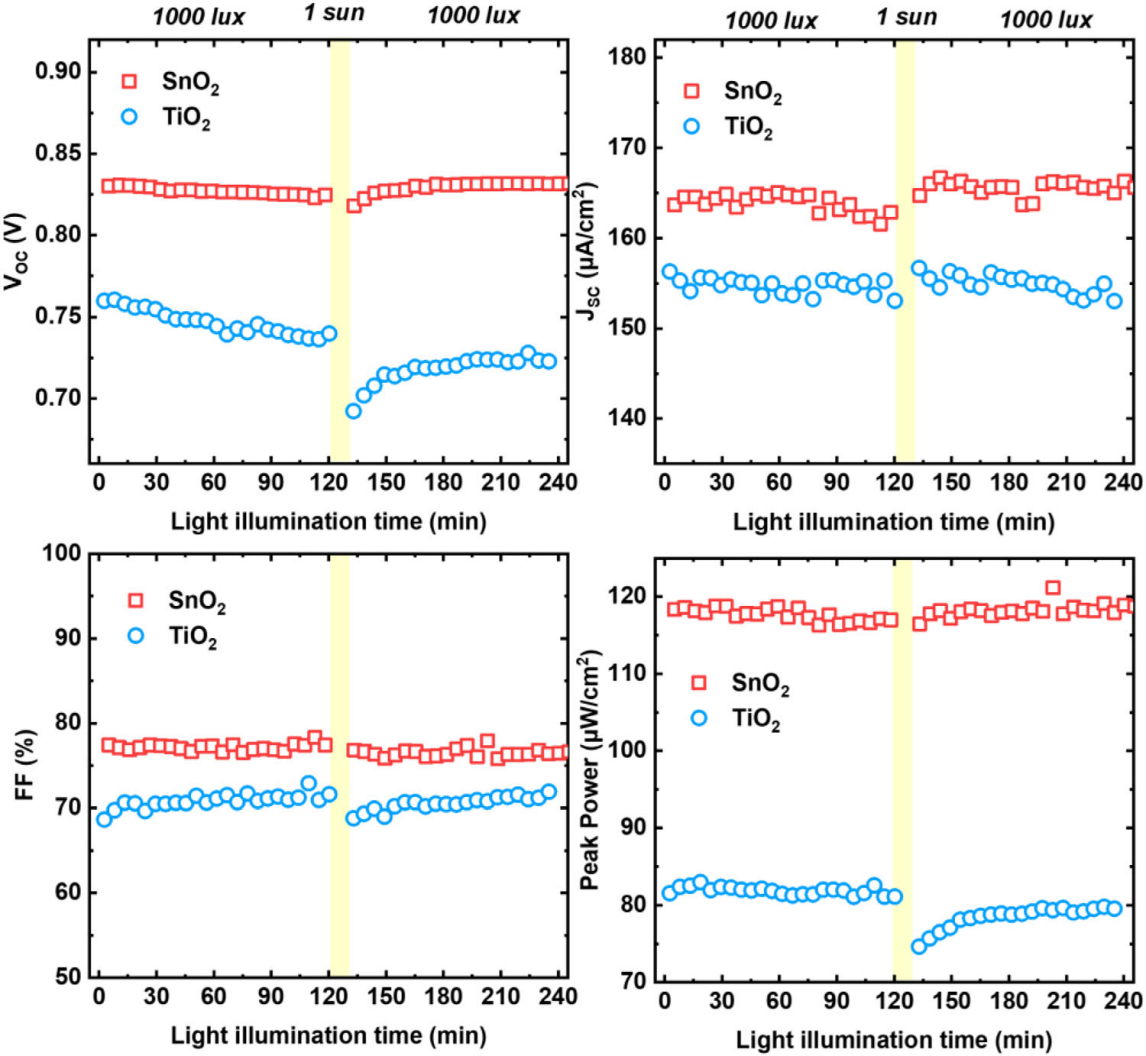
The Low‐High‐Low (LHL) measurements of devices using SnO_2_ and TiO_2_ ETLs.

The reduced V_OC_ in the TiO_2_‐based devices is consistent with increased interfacial recombination losses. In the initial J‐V measurement at 1000 lux, the carrier density is relatively low, resulting in the half‐filling of trap states. The V_OC_ drop in the TiO_2_ device during the initial low‐light illumination suggests a progressive interfacial recombination loss. Under 1 Sun, electron density is significantly increased and therefore trap states in the ETL are expected to be fully filled. These filled trap states can provide a pathway for photogenerated holes in the active layer to recombine. Under high‐light, the amount of filled trap states and photogenerated holes are both higher than under low‐light, leading to more significant interfacial recombination. When devices went back to low light, not all trapped electrons can be released from the trap states, and thus more filled trap states are kept acting as recombination centers than before 1 Sun illumination. The decrease right after 1 Sun illumination and slow recovery in the V_OC_ suggests that increasing interfacial recombination during the first step under 1000 lux is because trap states in the ETL are gradually filled (trapping), providing more recombination centres.

The smaller change in the V_OC_ of the SnO_2_ device after 1 Sun illumination, compared to that of the TiO_2_ device, illustrates that trap states filled under high‐light induce more interfacial recombination pathways and thus decrease V_OC_. This is consistent with the relative relationship between the Ideality Factor we measured under high‐light. Moreover, the carrier density under 1000 lux is not sufficient to fill all trap states, leading to V_OC_ recovery as trapped electrons release. Full recovery in the SnO_2_ device suggests a more stable interface and less trap states in the ETL, whereas incomplete recovery in the TiO_2_ device indicates possible irreversible degradation during the process of trapping and de‐trapping. This slow V_OC_ recovery in TiO_2_ is also consistent with the slow charge trapping and de‐trapping found in SPV measurements, further confirming the critical role of trap states in ETLs for charge carrier transport dynamics in devices. However, the origin of incomplete V_OC_ recovery in the TiO_2_ device is unclear, which is probably due to long‐lived trapped charge, ion migration, and possible chemical reactions at the interface [39, 40, 41]. Although the correlation between these irreversible processes and charge trapping/de‐trapping needs further investigation, this LHL measurement still reflects an unstable interface between the TiO_2_ ETL and the perovskite layer compared to the SnO_2_ ETL. This is consistent with the above results that significant photo‐induced potential change on TiO_2_ surface and interfacial trap‐assisted recombination loss at the TiO_2_/perovskite interface.

The long‐term stability of PSCs is an essential factor for such devices in reaching commercial viability. To evaluate the influence of interfacial recombination induced by oxide trap states in device stability, a 200‐h continuous light stability test was conducted in devices with SnO_2_ and TiO_2_ as ETLs under 1 Sun illumination in nitrogen (Figure 5). Experimental results show that SnO_2_‐based devices exhibited initial V_OC_, J_SC_, FF, and PCE of 1.1 V, 22 mA/cm^2^, 0.8, and 18%, respectively, which decreased to 0.9 V, 18 mA/cm^2^, 0.7, and 12% after 200 h, indicating moderate degradation. In contrast, TiO_2_‐based cells showed similar initial parameters (V_OC_ 1.1 V, J_SC_ 22 mA/cm^2^, FF 0.8, PCE 18%) but suffered severe degradation to 0.8 V, 15 mA/cm^2^, 0.6, and 8% after 200 h, with PCE dropping by 55.6% compared to SnO_2_’s 33.3%. J‐V curve analysis revealed minimal current loss and relatively stable V_OC_ retention for SnO_2_, while TiO_2_ displayed significant V_OC_ loss, suggesting faster degradation induced by intensified interfacial recombination. For both oxide ETLs, the FF dominates the performance loss mechanism, which is related to deteriorated charge extraction, suggesting that the interfacial trap states exert a great influence on performance degradation. The superior stability of SnO_2_ originates from its stable perovskite interface because it has less surface trap states, effectively suppressing photo‐induced degradation, whereas TiO_2_’s rapid decline correlates with trap‐assisted recombination and interfacial chemical instability. These findings demonstrate that SnO_2_ is more suitable for long‐term, commercial applications, while the stability of the perovskite/TiO_2_ interface is problematic.

**FIGURE 5:**
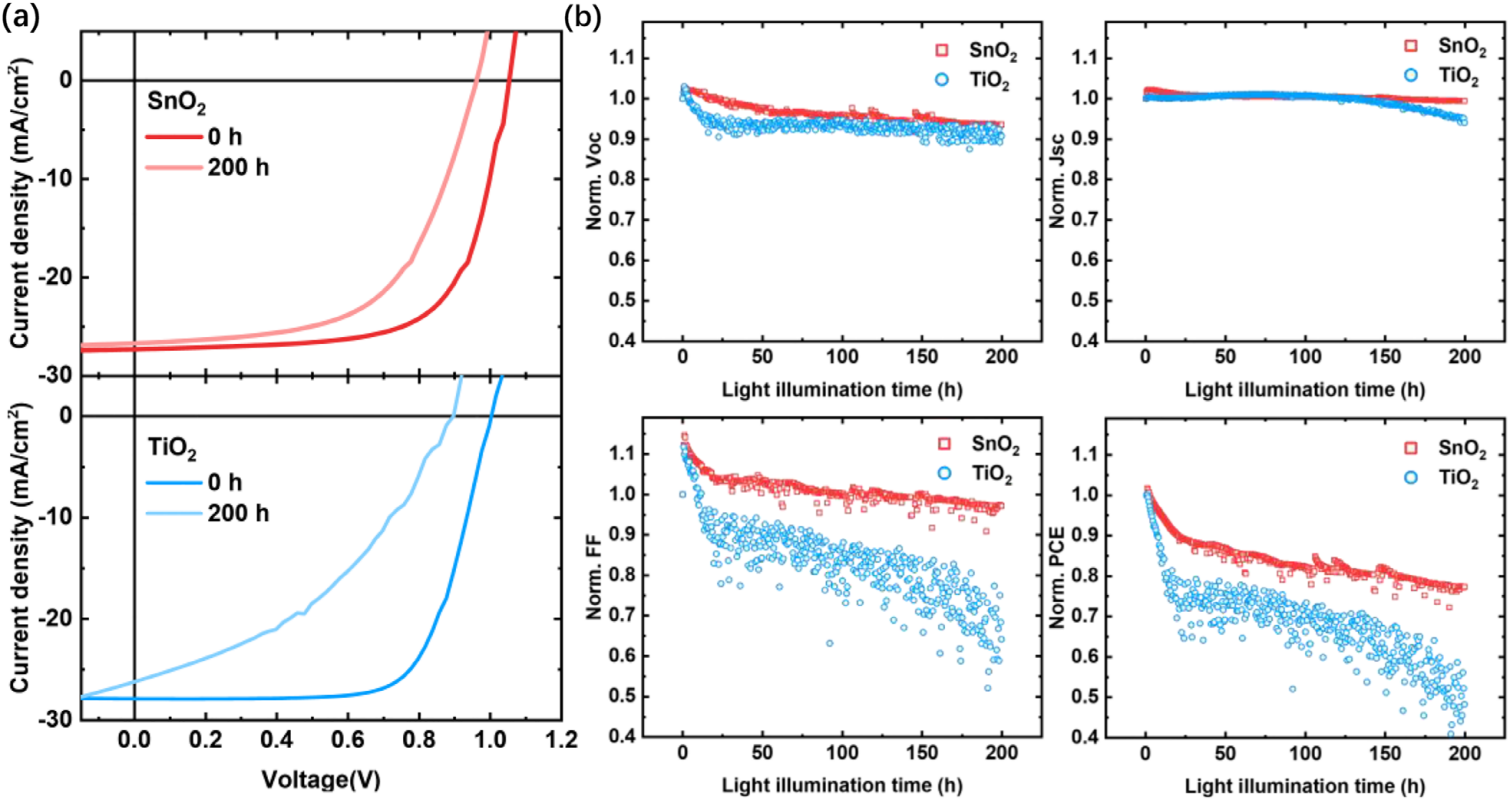
(a) Change in J‐V characteristics of SnO_2_‐ and TiO_2_‐based devices upon 200‐h measurement under 1 Sun. (b) V_OC_, J_SC_, FF and PCE evolution in the long‐term stability test.

### 2.3 Impact of ETL Trap States on Interfacial Charge Recombination

To compare the charge carrier dynamics influenced by the presence of different ETLs, we measured photoluminescence (PL) spectra and time‐correlated single photon counting (TCSPC) on the metal oxides. As shown in Figure 6a, the PL spectra of the perovskite deposited on SnO_2_ and TiO_2_ have peaks located at 770 nm, corresponding to the perovskite bandgap (∼1.61 eV), consistent with the perovskite absorption (Figure S8). The PL intensity of the perovskite on SnO_2_ is slightly lower than that on TiO_2_, indicating more efficient electron transport from the perovskite to the SnO_2_. For perovskite on ZnO, the PL peak is broadened and red‐shifted to 777 nm (Figure S9), which can be attributed to phase segregation [42]. The increase in PL intensity also suggests poor electron transport and instability at the interface between the mixed perovskite and ZnO.

**FIGURE 6:**
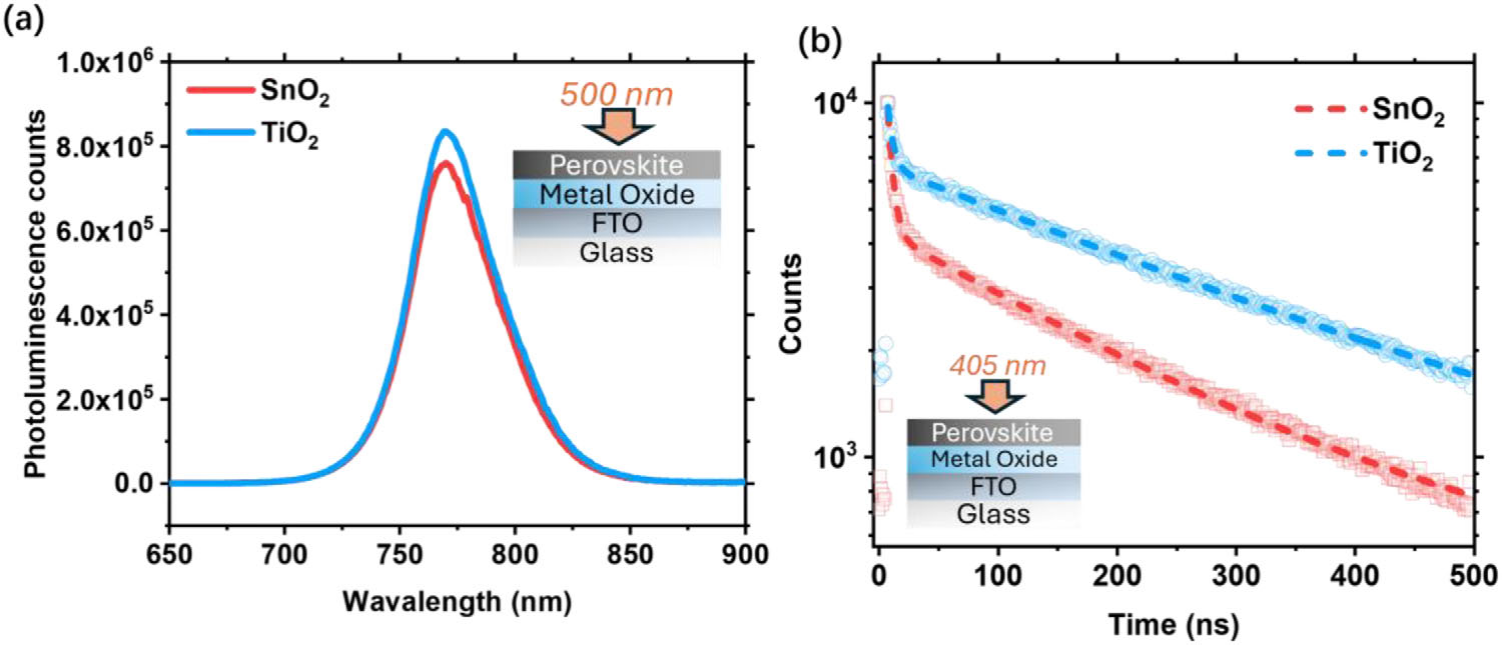
(a) Steady‐state photoluminescence (PL) spectra and (b) photoluminescence decays of the perovskite deposited on different metal oxide ETLs. The PL spectra were measured with 500 nm excitation in air. The decays were measured at 770 nm following 405 nm excitation in air.

To further confirm the charge‐transfer process between the perovskite layer and the ETL, the carrier lifetimes of these perovskite films were investigated by TCSPC. The photoluminescence decays of the perovskite films deposited on SnO_2_ and TiO_2_ at the emission peak of 770 nm are shown in Figure 6b. The fast‐decay component reflects charge transfer from the perovskite layer to the metal oxide ETL. The slow component reflects radiative recombination within the perovskite film. The decay traces fit well to a biexponential function, yielding fast (τ_1_) and slow (τ_2_) lifetimes, as listed in Table S2. In the case of SnO_2_, which has fewer trap states, the fast component is more significant, indicating more efficient electron transport from the perovskite layer to the SnO_2_ layer. Compared with SnO_2_, TiO_2_ has more trap states and extends the carrier lifetime in perovskite, suggesting that interfacial electron transport is hindered by these trap states. These results show that each oxide substrate imposes distinct interfacial charge dynamics, which depend on the amount of trap states on the oxide surface. The relative ordering of τ_1_ values matches the trend in relative amounts of trap states confirmed from SPV measurements. These findings indicate that metal oxide trap states control both charge extraction and recombination at the perovskite/oxide interface.

### 2.4 Impact of ETL Trap States on Device Performance—Simulation Results

To gain further insight into the influence of ETL traps on device performance across different light intensities, we simulated J‐V curves with varying interface trap densities. In this simulation, we consider a photovoltaic device containing interfacial traps at the ETL/perovskite interface and bulk traps throughout the active layer, where the recombination and occupation of the traps is described by Shockley‐Read‐Hall (SRH) statistics. Moreover, the interface traps are quantified using an effective interface recombination velocity (*v*_int_) scaling linearly with the interfacial trap density. Further details of the simulations are provided in the Methods.

Figure 7a shows a pronounced V_OC_ reduction under 1 Sun illumination with an increasing number of interfacial traps. In this case, photogenerated carriers dominate the carrier density, meaning that electron and hole densities become comparable. Increasing the interfacial trap density results in an enhanced band bending, induced by the trapped space charge, at the ETL/perovskite interface. This band bending substantially reduces the electric field in the active layer at high intensities, allowing for holes to more easily diffuse to the ETL interface near open‐circuit conditions. Given that trapped electrons can be recombined with photogenerated holes, a considerable amount of SRH recombination loss is subsequently induced by trap states which act as recombination centers. Under low light, the photogenerated carrier densities are small (dark electrons dominate near the ETL interface). In this case, the V_OC_ is smaller and well below the built‐in voltage, meaning that a strong electric field prevails in the active layer at these intensity levels. Because of the strong field, counteracting the screening induced by interfacial band bending, the photogenerated holes are driven away from the ETL. Subsequently, both the hole density and the SRH recombination through trap states are negligible at this interface (Figure 7b). As a result, under low light, the V_OC_ is mainly dominated by bulk SRH recombination and only affected by interface traps indirectly through their band bending at the interface, causing a partial reduction of the electric field within the active layer. At higher light intensities, however, the electric field (near open circuit) is weakened to such an extent that photogenerated holes and trap‐assisted recombination at the metal oxide ETL become significant. To summarize, the role of these metal oxide trap states is that they induce space charge effects and act as recombination centers at the interface. In this model, interface traps are most recombination‐active when hole density at the ETL interface rises, rather than simply because the total trap density is high.

**FIGURE 7:**
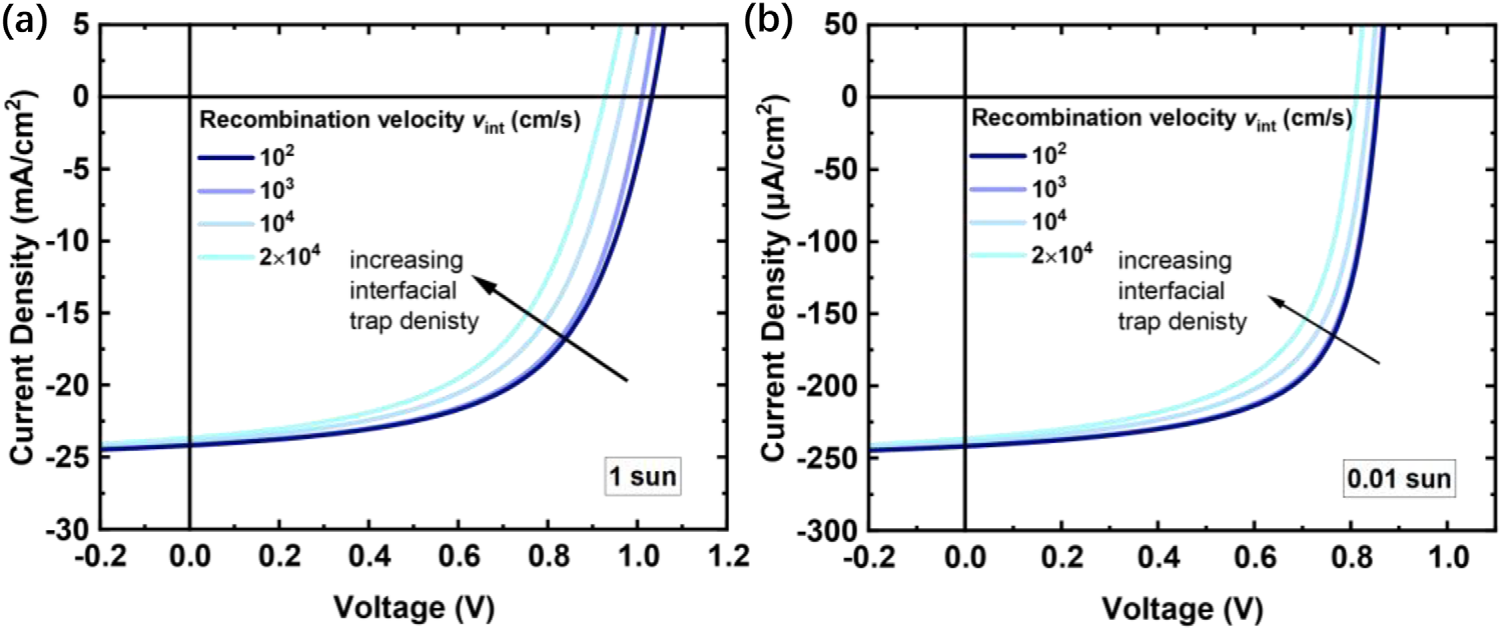
Simulated J‐V curves for equivalent light intensity conditions corresponding to (a) 1 Sun and (b) 0.01 Sun, with the latter being relevant for low‐light.

The light intensity‐dependent V_OC_ of devices with varying interfacial trap densities is also simulated. Under low light, the Ideality Factor approaches a value of *n* ∼ 1.7 as the recombination is dominated by bulk SRH recombination (as *n* ∼ 2). As the light intensity and thus the V_OC_ is increased, the screening effect of the interfacial trap density on the prevailing electric field increases. The recombination becomes dominated by recombination through interfacial trap states at the ETL/perovskite interface, as indicated by an Ideality Factor of *n* ∼ 1. As the space‐charge from the interface traps becomes significant, however, an Ideality Factor *n* < 1 is eventually obtained at high intensities as the electric field is completely screened and interfacial recombination dominates.

These simulation results show a strong agreement with our experimental results. Our SPV measurements suggest that SnO_2_ has less surface trap states than TiO_2_ (Figure 1), and the impact of these trap states on the device performance is shown in experimental J‐V curves (Figure 2), which correlates with simulated J‐V curves (Figure 7). Then light intensity‐dependent V_OC_ is shown in Figure 3, and it matches the simulation results (Figure 8). Noted that an Ideality Factor n < 1 are observed in both simulated and experimental light intensity‐dependent V_OC_, indicating the ETL with a large amount of surface trap states, such as untreated TiO_2_, leads to severe interfacial recombination. It is predicted that the effect of ETL trap states and interfacial recombination is stronger under high‐light, and much weaker under low‐light where bulk SRH becomes dominant. This matches with our light intensity dependent measurements of V_OC_ and J_SC_, where the difference between SnO_2_ and TiO_2_ ETL are more significant under high‐light but becomes smaller under low‐light. This is also consistent with the strong V_OC_ drop after the 1 Sun step in the Low‐High‐Low‐light measurements (especially for TiO_2_) and the partial recovery after returning to 1000 lux.

**FIGURE 8:**
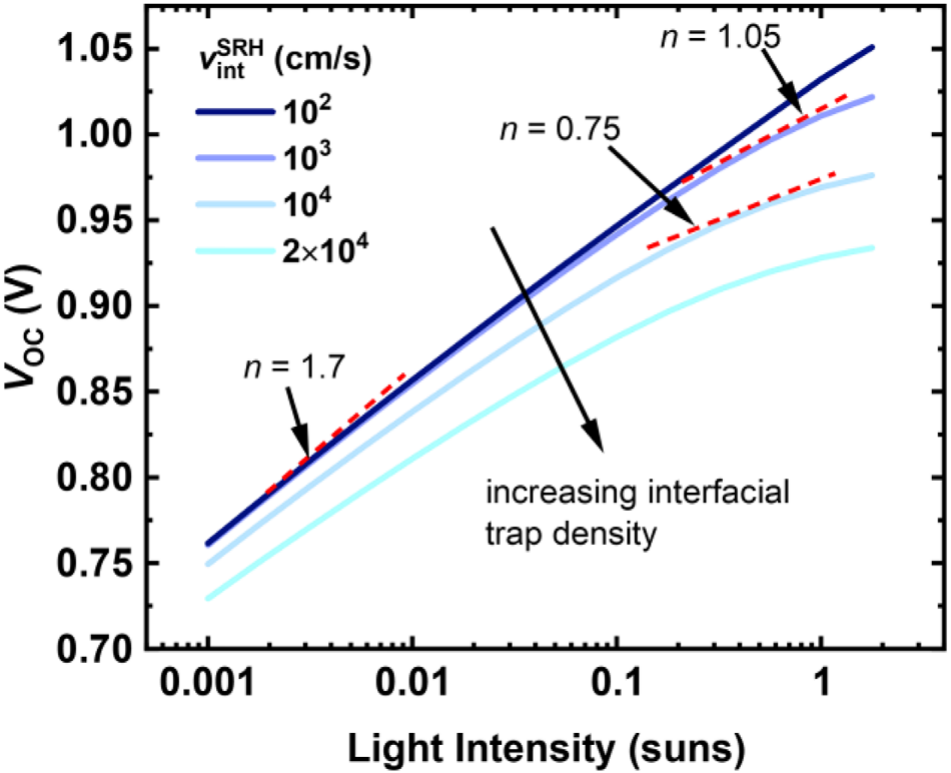
Simulated light intensity‐dependent V_OC_ of devices with varying interface trap densities.

## 3 Conclusion

These results demonstrate the importance of metal oxide ETL selection, and how their properties significantly affect both the V_OC_ and photostability of n‐i‐p planar perovskite solar cells. We show how changes in light intensity impact trap states introduced at the metal oxide/perovskite interface. Our experimental and simulation results show that high‐light leads to strong trapping/de‐trapping activities at the ETL/perovskite interface and significantly activates non‐radiative trap‐assisted recombination pathways, while trap states within the perovskite bulk become the dominant loss pathway under low‐light.

Metal oxide ETLs with higher density of traps induce larger photo‐induced surface potential changes, stronger interfacial recombination, greater V_OC_ loss, slower electron transport, and eventually poorer device stability. Our work links surface properties across metal oxide ETLs to device‐level energy loss mechanisms by revealing the illumination dependence of interfacial recombination. The findings have scientific significance and potential practical value for optimizing metal oxide/perovskite interfaces and designing devices for different illumination environments.

## 4 Methods

### 4.1 Preparation of Metal Oxides

For all oxide films, the cleaned substrates were treated with UV‐Ozone for 15 min (Ossila UV/O_3_ cleaner) immediately before deposition. SnO_2_ films were prepared by spin coating an aqueous colloidal SnO_2_ suspension (15 wt.%, Alfa Aesar) diluted to 2.67 wt.% at 4000 rpm. The deposited films were annealed in air at 140°C for 30 min. A compact TiO_2_ layer was deposited by spin coating a precursor solution containing titanium isopropoxide (0.22 M) and hydrochloric acid (0.03 M) in isopropyl alcohol at 2000 rpm. The films were dried in air at 150°C for 10 min and then annealed in a muffle furnace at 500°C for 30 min. ZnO films were prepared by a sol–gel method. Zinc acetate dihydrate and 2‐aminoethanol were dissolved in 2‐methoxyethanol at equimolar concentration (0.3 M), followed by overnight sonication to obtain a homogeneous precursor solution. The ZnO precursor was spin‐coated onto the substrate, and the films were then annealed in air at the desired temperature 200°C for 45 min.

### 4.2 Preparation of Perovskite

The perovskite composition used in this work was Cs_0.05_Ma_0.14_FA_0.81_Pb(I_0.86_Br_0.14_)_3_. The precursor solution was prepared in DMF/DMSO (4:1, v/v) and deposited in a nitrogen‐filled glovebox. The solution was spin‐coated using a two‐step program: 1000 rpm for 10 s, followed by 4000 rpm for 30 s. Chlorobenzene (110 µL) was dropped onto the spinning substrate 10 s before the end of the second step as the antisolvent. The films were then annealed at 100°C for 30 min.

### 4.3 Preparation of Spiro‐HTL

The Spiro‐OMeTAD solution consisted of 73 mg mL^−1^ Spiro‐OMeTAD in chlorobenzene, with *t*‐BP, Li‐TFSI solution (500 mg mL^−1^ in acetonitrile), and Co‐TFSI solution (400 mg mL^−1^ in acetonitrile) as additives.

### 4.4 Device Fabrication

FTO substrates for device fabrication were sequentially cleaned by sonication in acetone, detergent solution, and ethanol for 15 min each. The metal oxide ETLs were then deposited onto the cleaned FTO substrates as described above, followed by deposition of the perovskite layer. After annealing of the perovskite films at 100°C for 30 min and cooling to room temperature, the hole‐transport layer was deposited by spin coating a Spiro‐OMeTAD solution at 3000 rpm for 30 s. All devices were finished with a 70 nm Au top electrode, with a shadow mask defining a pixel area of 0.045 cm^2^ each. The complete device structure is Glass/FTO/Metal oxides/perovskite/Spiro/Au.

### 4.5 Characterization of PSCs

Current density‐voltage (J‐V) curves were collected using a Keithley 2400 with the devices exposed to 1 Sun illumination (Wacom WXS‐156S‐10 Solar Simulator). A scan speed of 0.1 V/s was used, with the devices being first being scanned in the forward direction from rest before being allowed to return to rest and scanned in the reverse direction. For the Low‐High‐Low (LHL) measurement, each device was sequentially exposed to 1000 lux for 2 h, 1 Sun for 10 min, and 1000 lux for a further 2 h. J‐V characteristics were measured throughout the cycle, and the evolution of V_OC_, J_SC_, FF, and PCE was tracked. Stability tests were carried out using a custom LED solar simulator with an intensity of 1000 W m^−2^ and approximate AM1.5G spectral match.

### 4.6 Surface Photovoltage

Surface Photovoltage (SPV) measurements were conducted by a Kelvin Probe. The contact potential difference (CPD) was collected, and SPV was recorded by tracking the change in CPD upon illumination. A lamp bulb with max intensity of 1/5 Sun was used as the light source.

### 4.7 Time‐Correlated Single Photon Counting

Time‐correlated single photon counting (TCSPC) measurements were collected using an Edinburgh Instruments FLS1000 fitted with a 405 nm excitation laser (6 mW cm^−2^ at 2 MHz). A long‐pass filter (>455 nm) was fitted between the sample and the detector to reduce the impact of reflected excitation light.

### 4.8 Simulations

For the simulations, a 1D drift‐diffusion model was employed. The model numerically solves the Poisson equation and the electron and hole continuity equations, assuming the current densities to be governed by drift and diffusion. Numerical details of the device model are provided in Ref. [43]. In the simulations, we assume a generic n‐i‐p perovskite device of the form: cathode/ETL/perovskite active layer/HTL/anode. The HTL (Spiro‐OMeTAD) is assumed to be highly doped, effectively acting as part of the anode contact. A bandgap of 1.6 eV is assumed for the perovskite, forming a conduction band offset of 0.2 eV at the ETL/perovskite interface. For simplicity, we assume uniform photogeneration rates and that the effect of ions is negligible. The recombination of electrons and holes in the active layer is assumed to take place both via bimolecular recombination and recombination via deep trap states. The trap states are described by Shockley‐Read‐Hall (SRH) statistics and assumed to be present both at the ETL/perovskite interface and throughout the bulk of the perovskite active layer. Furthermore, the SRH recombination via the interface traps, taking place between holes in the perovskite active layer and electrons in the ETL, is represented using an effective interface recombination velocity (*v*_int_) which is directly related to their interfacial trap density (*N*_int_) as *v*_int_ = *c*_int_ *N*_int_. Here, *c*_int_ is the capture coefficient for interface traps, assumed to be equal for electrons and holes, and given by *c*_int_ = 8 × 10^−8^ cm^3^/s.

## Author Contributions

**Tianhao Lan**: conceptualization, Writing – review and editing, project administration, resources, validation, methodology, Writing – original draft. **Joel Luke**: conceptualization, methodology, writing – review and editing. **Oskar J. Sandberg**: investigation, writing – review and editing, methodology. **Charlie Henderson**: writing – review and editing, validation. **Ying Sun**: validation, investigation. **Katherine Stewart**: writing – review and editing, validation. **Ji‐Seon Kim**: conceptualization, funding acquisition, writing – review and editing, project administration, supervision, resources, validation, methodology, writing – original draft.

## Conflicts of Interest

The authors declare no conflicts of interest.

## Supporting information




**Supporting File**: advs78501‐sup‐0001‐SuppMat.docx.

## Data Availability

The data that support the findings of this study are available from the corresponding author upon reasonable request.
