# Supplementary material for: Illumination‐Dependent Interfacial Recombination in Perovskite Photovoltaics Driven by Trap States in Metal Oxide Electron Transport Layers
Source: Adv Sci (Weinh). 2026 Oct 30:e78501. Online ahead of print. doi: 10.1002/advs.78501 (PMC13721585; doi:10.1002/advs.78501)
Supplement: Supplementary file 1 [file ADVS-9999-e78501-s001.docx]

Supporting Information

**Illumination-Dependent Interfacial Recombination in Perovskite Photovoltaics Driven by Trap States in Metal Oxide Electron Transport Layers**

Tianhao Lan,^1^ Joel Luke^1,2^, Oskar J. Sandberg^3^, Charlie Henderson,^1,4^ Ying Sun,^1^ Katherine Stewart,^1^ and Ji-Seon Kim^1,4,5*^

1. Department of Physics and Centre for Processable Electronics, Imperial College London, London, UK
2. Physical Sciences and Engineering Division (PSE), King Abdullah University of Science and Technology (KAUST), Thuwal, 23955–6900 Saudi Arabia
3. Department of Physics, Faculty of Science and Engineering, Åbo Akademi University, Turku, Finland
4. Department of Chemistry, University of Oxford, South Parks Road, Oxford, OX1 3QZ, United Kingdom
5. Department of Physics, Ewha Womans University, Seoul 03760, Republic of Korea

Email: ji-seon.kim@chem.ox.ac.uk

Table S1 The detailed device performance parameters of perovskite solar cells with different metal oxide ETLs under 1 Sun and 1000 lux.

| **Light intensity** | **ETL materials** | **V_OC_ (V)** | **J_SC_ (mA cm^-2^)** | **FF (%)** | **PCE (%) / P_out_ (μW cm^-2^)** |
| --- | --- | --- | --- | --- | --- |
| 1 Sun | SnO_2_ | 1.01±0.03 | 22.4±0.9 | 67.6±4.0 | 15.3±1.2 |
|  | TiO_2_ | 0.96±0.04 | 20.6±0.8 | 74.3±3.6 | 14.8±1.4 |
|  | ZnO | 0.89±0.08 | 23.3±1.0 | 63.6±8.2 | 13.2±2.8 |
| 1000 lux | SnO_2_ | 0.83±0.02 | 0.164±0.008 | 70.6±8.0 | 95.8±13.3 |
|  | TiO_2_ | 0.79±0.05 | 0.161±0.005 | 71.1±6.9 | 91.4±14.1 |
|  | ZnO | 0.76±0.08 | 0.161±0.008 | 63.1±7.2 | 78.8±19.0 |

Table S2 Summaries of fitting parameters in TCSPC for perovskite deposited on different oxide ETLs. The metal oxide with less trap states shows the shorter charge lifetime, indicating a more efficient electron transport.

| **ETL materials** | **τ₁ (ns)** | **τ₂ (ns)** | **τ₁/ τ₂** |
| --- | --- | --- | --- |
| SnO_2_ | 4.46±0.08 | 210.01±2.73 | 0.021 |
| TiO_2_ | 4.46±0.13 | 303.32±3.95 | 0.015 |
| ZnO | 8.04±0.61 | 667.24±38.97 | 0.012 |

(a)

(b)

Figure S1 (a) The spectrum of white light for SPV measurements. (b) The power of each LED light for wavelength-dependent SPV measurements.

(a)

(b)

(c)

(d)

(e)

(f)

Figure S2 Comparison of SPV measurements under vacuum and in the air of (a) SnO_2_, (b) TiO_2_ and (c) ZnO. The first cycle of normalised SPV measurements under vacuum and in the air of (d) SnO_2_, (e) TiO_2_ and (f) ZnO.

The SPV response of the metal oxides was measured under high vacuum to investigate the impact of surface adsorbate or reactions with oxygen in the air. As shown in Figure S2, both the SnO_2_ and TiO_2_ exhibit increased SPV magnitude under HV compared to atmosphere (Figure. S2), with the SPV response of TiO_2_ (-559 mV at the end of the second light cycle) significantly greater than SnO_2_ (-145 mV). This is attributed to the dual effect of the volatilisation surface adsorbates (e.g. water) at HV and the generation of more oxygen vacancies at the oxide surface. Surface adsorbates such as water are known to passivate metal oxide oxygen defects.^1^ When moving to HV, surface adsorbed water will volatilise, reactivating passivated trap states and leading to increased SPV magnitude. As oxygen vacancy formation energy depends on O_2(g)_ partial pressure at the interface,^2-3^ moving from atmosphere to HV will also result in an increase in the density of oxygen vacancies (i.e. traps) at the surface. These results demonstrate that atmosphere in which SPV measurements are conducted must be considered when reporting the absolute magnitude of an SPV response. However, as the trend in SPV magnitude of the TiO_2_ and SnO_2_ samples is the same in both atmosphere and HV, our conclusion that there is a greater density of surface trap states in TiO_2_ is unaltered by these experiments. The results of the same experiment carried out on ZnO is included below for completeness. Unexpectedly, ZnO exhibits similar SPV magnitude under HV conditions and atmospheric pressure; however, significant persistent SPV is observed following illumination. This may be indicative of changes in de-trapping rate caused by a change in the nature of the defect states, resulting in charges being trapped persistently at the surface. Fully understanding this interesting behaviour is out of the scope of this study and could form the basis of further investigation.

Figure S3 The energy band schematic of metal oxide measured under sub-bandgap excitation.

Under sub-bandgap excitation, direct valence-band-to-conduction-band excitation is not expected. A measurable SPV response therefore indicates that defect levels within the band gap are involved. Filled defect states can be photoionized into the conduction band, which changes trap occupancy and modifies the surface charge, leading to a change in band bending. Therefore, we measured the SPV of metal oxides under sub-bandgap excitation to investigate trap-related charge dynamics. The negative SPV observed under sub-bandgap excitation confirmed the photoionization of occupied defect states into the conduction band. In this process, sub-bandgap photons release trapped electrons into the conduction band and leave behind positively charged states, leading to the reduction of upward band bending. Here, the sub-bandgap SPV response mainly reflects trap occupancy changes and electron redistribution rather than direct band-to-band photogeneration, but it should not be considered as an absolute measure of total trap density since the band bending is affected by many factors.

(a)

(b)

Figure S4 (a) SPV measurements of the perovskite layer on different metal oxide ETLs. The negative SPV signal indicates hole accumulation on the top surface of perovskite, further confirming the n-type conducvity and good electron extration ability of these metal oxides. (b) Energy levels of different metal oxide ETLs. The Fermi levels are measured by APS system, while the CBM and VBM are collected from literatures.^4-6^

For perovskite deposited on metal oxides, SPV no longer probes only the free oxide surface. It probes the whole half-stack response from the metal oxide/perovskite interface, perovskite absorber, top perovskite surface. Usually, a larger SPV of perovskite with a transport layer indicates a better charge extraction, but here the contribution of the interfacial trapping is not negligible. Compared to Figure 1a, the change of the SPV shown in Figure S2a suggests that the contribution to charge redistribution from electron extraction at oxide/perovskite interface. The larger SPV enhancement of SnO_2_/perovskite and TiO_2_/perovskite (250 mV) indicate a good electron transport at the buried interface. In contrast, the smaller change of SPV in ZnO/perovskite (150 mV) suggests stronger charge trapping at the buried interface, which is indicated from the SPV of bare metal oxides and consistent with its poor performance in complete devices.

(a)

(b)

(c)

(d)

*1 Sun*

*1 Sun*

*1000 lux*

*1000 lux*

Figure S5 Comparison of (a) J-V characteristics and (b) statistical data of the average device using the ZnO ETL with SnO_2_ and TiO_2_ ETL under 1 Sun and (c, d) 1000 lux.

Figure S6 XRD of the perovskite layer coated on different metal oxides. No obvious peak change observed in this measurement, indicating that the morphology and composition of perovskite have not been influenced by oxide ETLs.

(a)

(b)

Figure S7 (a) The ambient photoemission spectra (APS) and density of states (DOS) spectra of the perovskite on different metal oxide ETLs. The perovskites on SnO_2_ and TiO_2_ show the same APS and DOS spectra, suggesting that the morphology and composition of perovskite have not been influenced by these two oxides.

(a)

(b)

Figure S8 (a) Transmittance and (b) absorbance of the perovskite film on different metal oxdie ETLs. No obvious change observed within 300 to 700 nm.

Figure S9 Steady-state photoluminescence (PL) spectra of the perovskite deposited on ZnO, showing a red-shifted peak position compared to that on SnO_2_ and TiO_2_.

(a)

(b)

(c)

**RMS = 29.6 nm**

**RMS = 23.7 nm**

**RMS = 24.5 nm**

Figure S10 Atomic force microscopy (AFM) images and root mean square (RMS) roughness of the surface of (a) SnO_2_, (b) TiO_2_ and (c) ZnO deposited on FTO.

**References**

1. J. S. Heo, J. W. Jo, J. Kang, et al., "Water-Mediated Photochemical Treatments for Low-Temperature Passivation of Metal-Oxide Thin-Film Transistors," *ACS Applied Materials & Interfaces* 8, 16, (2016): 10403, https://doi.org/10.1021/acsami.5b12819.

2. Q. Hou, J. Buckeridge, T. Lazauskas, et al., "Defect formation in In_2_O_3_ and SnO_2_: a new atomistic approach based on accurate lattice energies," *Journal of Materials Chemistry C* 6, 45, (2018): 12386, https://doi.org/10.1039/c8tc04760j.

3. A. Janotti, J. B. Varley, P. Rinke, et al., "Hybrid functional studies of the oxygen vacancy in TiO_2_," *Physical Review B* 81, 8, (2010), https://doi.org/10.1103/PhysRevB.81.085212.

4. H. Luo, S. Dimitrov, M. Daboczi, et al., "Nitrogen-Doped Carbon Dots/TiO_2_ Nanoparticle Composites for Photoelectrochemical Water Oxidation," *ACS Applied Nano Materials* 3, 4, (2020): 3371, https://doi.org/10.1021/acsanm.9b02412.

5. Z. Li, R. Wang, J. Xue, et al., "Core-Shell ZnO@SnO_2_ Nanoparticles for Efficient Inorganic Perovskite Solar Cells," *Journal of the American Chemical Society* 141, 44, (2019): 17610, https://doi.org/10.1021/jacs.9b06796.

6. A. Sharma, S. E. Watkins, G. Andersson, D. A. Lewis, "Effect of Annealing Temperature of ZnO on the Energy Level Alignment in Inverted Organic Photovoltaics (OPVs)," *Energy Technology* 2, 5, (2014): 462, https://doi.org/10.1002/ente.201300186.
